# Predictors of patient safety activities among registered nurses and nurse aides in long-term care facilities: cross-sectional study

**DOI:** 10.1186/s12877-022-03234-w

**Published:** 2022-06-29

**Authors:** Youran Lee, Eunhee Cho

**Affiliations:** 1 Duke University School of Nursing, 307 Trent Drive, Durham, NC 27710 USA; 2 Mo-Im Kim Nursing Research Institute, Yonsei University College of Nursing, 50-1 Yonsei-ro, Seodaemoon-Gu, Seoul, 03722 South Korea

**Keywords:** Patient safety, Registered nurses, Nurse aides, Long-term care

## Abstract

**Background:**

In Korea, nurse aides (NAs) are legally permitted as substitutes for registered nurses (RNs) in long-term care (LTC) facilities, even though they have very different levels of education and qualification standards. Many studies in hospitals have shown better hospital nurse staffing, more educated nurses, and improved nurse work environments have been associated with lower hospital mortality and length of stay. There is research showing that a higher percentage of RNs with a bachelor’s degree corresponded to lower incidence rates of pressure ulcers in Korean LTC facilities. This study aimed to explore the factors that influence patient safety activities of the RNs and NAs working in LTC facilities and to identify the relationship between patient safety culture (PSC) and patient safety activities.

**Methods:**

This study is a descriptive cross-sectional survey. The study participants were conveniently collected from 88 RNs and 71 NAs who worked at 33 LTC facilities for more than three months. The patient safety activities tool was developed by the researchers for residents of LTC facilities based on the tools developed by Park et al. (2012) for hospital nurses and the patient safety goals of the Joint Commission. The questionnaires were collected by email or mobile application and kept confidential. Data were analyzed using descriptive statistics, independent t-test, one-way ANOVA, Pearson correlation coefficients, and multiple regression analysis.

**Results:**

The mean scores of PSC and patient safety activities were 4.03 ± 0.51 points and 4.29 ± 0.49 points out of 5, respectively. There was significant correlation between PSC and patient safety activities (r = .23, *p* = .004). Factors influencing patient safety activities among RNs and NAs in LTC facilities were RNs (*β* = .377, *p* < .001), organizational system of PSC (*β* = .314, *p* < .010), and work shift type (fixed night shift, on-call, 24-h shift) (*β* = -.264, *p* = .004), which explained about 36.0% of total variance (F = 5.69, *p* < .001).

**Conclusion:**

The findings indicate that it is necessary to mandate RNs instead of NAs to enhance residents’ safety in LTC facilities. Additionally, the importance of an organizational safety system and effective working shift types to prevent residents’ safety accidents in LTC facilities is indicated.

**Supplementary Information:**

The online version contains supplementary material available at 10.1186/s12877-022-03234-w.

## Background

Korea’s aging rate is the fastest in the world [1]. In addition to the aging population, female economic participation and the nuclearization of families have made it challenging to provide care for older people with general weakness and chronic diseases. In response, the Korean government assumed the responsibility for addressing problems related to these older adults and enacted and enforced a long-term care (LTC) insurance system in July 2008 [2]. The average number of beds in LTCs is 55 [3]. For facilities covered by LTC insurance, the government stipulates that at least one doctor, oriental medicine doctor, or dentist must contract with LTC facilities of any size. This provider visits the contracted LCT facility once every two weeks. In addition, over 60% of Korean LTC facilities have no RN [4]. Therefore, most residents who require medical treatment are transferred to acute hospitals. LTC residents often display behavioral and cognitive disorders, such as dementia, delusion, depression, anger, and aggression; most cannot conduct their daily lives independently for more than six months, highlighting the need for intensive management of safety incidents among older adults in LTC facilities [5]. Although the incidence of adverse events at LTC facilities has not been reported in Korea, 84.2% of LTC facility staff have experienced a safety incident [6], indicating the seriousness of safety risks for the older adults in LTC facilities.

There are no legal grounds for specifying the staffing level, role regulation, and direct nursing time in LTC facilities in Korea. The existing legal stipulation for nursing staffing in LTC facilities requires one RN or a nurse aide (NA) per 25 residents with more than 30 beds, and one RN or NA with 10–30 beds [7]. However, the wide gap between the levels of education and licensure for an RN and NA presents a problem. While RNs complete a four-year curriculum at a university and obtain a license if they pass the government examination, NAs complete a one-year program from an educational training institution and obtain a certificate if they pass the government examination. However, RNs and NAs are expected to practice similar roles in LTC facilities because of insufficient regulations. In 2019, there were 1.75 persons employed as nursing staff per LTC facility in Korea, of which only 0.28 persons were RNs [8, 9]. The quality of care is closely related to the standards and levels of staffing. Many studies in hospitals have shown RN staffing, a higher percentage of RNs with a bachelor’s degree, and improved nurse work environments have been associated with lower hospital mortality and length of stay [10–12]. According to the research conducted in LTC facilities, more RNs influence fewer incidences rate of aggressive behavior, depression, weight loss, and bed rest [13]. Furthermore, one study found that Korean LTC facilities employing a higher percentage of RNs with a bachelor’s degree reported lower incidence rates of pressure ulcers [14]. The vulnerable staffing structure in LTC facilities can have numerous adverse effects on the safety of their older adults; the influences of RN and NA staffing levels should be also considered.

Other countries (e.g., the United States, Canada, England, and Germany) have long been discussing the quality management of LTC facilities as a major policy issue [15]. Based on the literature examining the quality of LTC facilities, these countries report that problems such as falls, pressure ulcers, infections, medication errors, and malnutrition in the resident population of these facilities are preventable and are associated with nursing staff shortages [16]. Particularly, high staffing standards and staffing levels in RNs have been linked to positive outcomes such as improved quality of service delivery and reduced hospitalization rates among older adults in care facilities [17–19]. In the United States, each state has its own minimum staffing standards for RNs and licensed practical nurses/licensed vocational nurses in LTC facilities [20]. Further, these standards present the daily minimum nursing service time by RNs, licensed practical nurses/licensed vocational nurses, and clinical nurse aides per resident and recommend that 30% of such nursing services should be provided by an RN and that a nurse should be on duty 24 h a day [21].

Patient safety experts emphasize that structural problems related to the organizational system, compared to an individual’s error or indifference, are the more important causes of error, and they recommend ameliorating the safety system in work environments and establishing a PSC in order to prevent errors [22]. Nurses play an especially important role in identifying and managing risk to ensure patient safety at LTC facilities. PSC is commonly believed to promote patient safety activities and has a positive impact on safety outcomes [23]. Organizations with a positive PSC stress the importance of organizational policies, systematic processes, leadership that emphasizes patient safety, teamwork through efficient communication, efficient staffing allocation, and a reporting system for medical malpractice [24]. Many studies on PSC observed that better perceptions of PSC among nursing providers had a greater positive impact on patient safety activities [25]. However, the perception of PSC among LTC facility employees was worse overall than that of hospital employees [5]. Additionally, the perception of PSC varies depending on the staff in LTC facilities [26].

Previous studies have investigated employees’ perceptions of PSC in LTC facilities [5, 27–30], but research on the relationship between the perceptions of PSC and patient safety activities of nursing staff is inadequate. To date, no study has attempted to investigate the relationship between the perception of PSC and patient safety activities among RNs and NAs who are responsible for the care and safety of LTC facility residents, especially regarding legislation allowing NAs to function as RNs irrespective of their qualifications. This study aimed to identify the relationship between PSC and patient safety activities and to explore the factors that influence patient safety activities of the RNs and NAs working in LTC facilities.

## Methods

### Study design

This study is a descriptive cross-sectional survey aimed at identifying the relationship between PSC and patient safety activities and to explore the factors that influence patient safety activities in LTC facilities.

### Participants

RNs and NAs who have been working in a LTC facility for at least three months and provided informed consent to participate in this study were enrolled. Those who did not provide direct care or had less than 3 months of working experience at current facility were excluded from the study. Sample size was calculated utilizing G*power 3.1.9.4 software for regression analysis with a significance level (α) of 0.05, moderate effect size of 0.15, power (1-β) of 0.80, and 16 independent variables with reference to previous studies. The minimum required sample size was 143. We included data from 159 people in 33 facilities in the final analysis.

### Data collection and ethical consideration

The study was approved by the Institutional Review Board of the Y University Health System (Approval No. Y-2019–0096, dated 28 Aug 2019). Data were collected from RNs and NAs who work in LTC facilities from October 8 to October 31, 2019. We conveniently selected the LTC facilities from the nationwide National Health Insurance Service (NHIS) data on 3,390 LTC facilities after classifying the LTC facilities as < 100 beds and ≥ 100 beds to reduce the impact of differences in the size of facilities on results. We called the heads of the selected LTC facilities to explain the purpose and contents of the study and obtained their permission to collect data. The research manual was sent to the facilities that allowed data collection. When RNs and NAs who wanted to participate in the study contacted the researcher, the UTL address was transmitted by email or mobile phone, and the participants were included in the study. Due to the small number of subjects in one facility, more facilities were randomly added from the database until suitable subjects were obtained for the study. We obtained written and informed consent from all participants. The questionnaires were collected by email or mobile application and kept confidential.

### Instruments

#### Characteristics of individuals and facilities

The individual and facility characteristics that were found to affect nursing personnel's perception of patient safety culture and patient safety activities were reflected as study variables. As individual characteristic factors, age, marital status, education level, working experience at current facility (years), type of work shift, experience in reporting safety incident, and safety education status were considered. For LTC facilities, the number of facility residents, number of residents in charge, facility evaluation grade, type of facility, and proportion of RNs were investigated.

#### Patient safety culture

The perception of patient safety in LTC facilities was assessed using the Korean Patient Safety Culture Scale for LTC facilities developed by Yoon and Wu [6]. This 27-item scale consists of four factors: manager’s leadership (nine items), work attitude (six items), organizational system (seven items), and managerial practice (five items). Each item was rated on a five-point Likert scale from “strongly disagree” (1) to “strongly agree” (5); a higher score indicated a higher perception of PSC. The reliability of the scale was .95 in the study by Yoon and Wu [6], and .84 in our study.

#### Patient safety activities

The patient safety activities tool was developed by researchers for residents of LTC facilities based on the tools developed by Park et al. [31] for hospital nurses and the patient safety goals of the Joint Commission [32]. The scale of Park et al. (2012) [31] was composed of a 72-item scale consisting of nine factors: falls (twelve items), education (five items), infection (ten items), facility check (one item), fire safety (four items), patient identification (six items), communication (four items), medication (fourteen items), and blood transfusion (sixteen items). Each item was rated on a five-point Likert scale from “never practice” (1) to “always practice” (5); a higher score indicated a higher patient safety activities score. Among the nine factors, only patient safety activities performed in LTC facilities were selected. Therefore, blood transfusion was removed from the final patient safety activities tool. In addition, the tool was revised based on feedback from interviews with RNs and NAs in LTC facilities. To test the content validity of the tool, we formed a patient safety expert panel comprised of four nursing professors and three geriatric nurse practitioners working in LTC facilities. This panel rated the validity of each item for measuring the properties of patient safety activities in LTC facilities using a content validity checklist based on a scale consisting of “very relevant (4),” “quite relevant (3),” “somewhat relevant (2),” and “not relevant (1).” The content validity index (CVI) for each item was computed based on the criterion suggested by Lynn [33], and items with a CVI of 0.8 or higher were selected. Based on this restriction, all 41 items were selected. Patient safety activities consisted of five domains: safe medication administration (eight items), infection prevention (12 items), fall prevention (13 items), pressure ulcer prevention (five items), and facility inspection and fire safety education (three items). Each item was rated on a five-point Likert scale from “strongly disagree” (1) to “strongly agree” (5), with a higher score indicating a higher compliance with patient safety activities in the corresponding domain. The reliability of the entire tool was 0.83 in our study, with 0.79 for safe medication administration, 0.78 for infection prevention, 0.77 for fall prevention, 0.79 for pressure ulcer prevention, and 0.82 for equipment/fire inspection.

### Data analysis

The collected data were analyzed using SPSS Windows 25.0 (IBM Corp. Armonk, NY, USA) software. First, participants’ general characteristics and level of PSC and patient safety activities were analyzed with descriptive statistics. Second, variations in patient safety activities according to general organizational characteristics and patient safety characteristics were analyzed using independent t-tests, one-way ANOVA, followed by Scheffé post hoc test. Third, the relationship between PSC and patient safety activities was analyzed by calculating Pearson’s correlation coefficient. Fourth, the predictors of patient safety activities were identified using multiple regression analysis, including the personal characteristics of RNs and NAs, characteristics of LTC facilities, and patient safety culture.

## Results

### Descriptive analysis of participants and facilities

Participants’ demographic characteristics, work-related characteristics, and organizational characteristics are shown in Table 1. In total, 159 participants were enrolled, comprised of RNs (55.3%) and NAs (44.7%). The vast majority were women (98.7%), and the mean age was 50.92 ± 8.47 years. There were more single (54.1%) participants than married (45.9%), and the most common education level was a bachelor’s degree (32.7%). The most common working experience length at their current facility was between one and five years (34.6%). The most common work shift type was three shifts (42.8%), followed by fixed day shift (27.7%), and other (either fixed night shift, on-call, 24-h shift; 16.4%). In all, 73.0% of the participants had reported a safety incident within the last year, and the most common type of reported safety incidents were falls (68.6%), followed by pressure ulcer (37.7%), medication error (15.7%), and burns (8.2%). The vast majority (93.7%) completed a safety incident prevention course, included in safety nursing activities, within the past year. Regarding organizational characteristics, the most common facility admission capacity was 100–199 (40.3%), and the mean number of residents assigned per RN or NA was 75.54, with a range of 10–296. Regarding the LTC facility evaluation ratings, the majority (74.8%) received an A rating, followed by a B rating (12.6%) and C rating (12.6%). The most common type of LTC ownership was a foundation (34.0%), followed by public (33.3%) and private (32.7%). Comparing RNs and NAs, the most common proportion of RNs in the nursing staff was 50–74% (47.2%).

1. Perception of PSC and level of patient safety activities
2. The participants’ perceptions of PSC and level of patient safety activities are shown in Table 2. The mean perception of PSC was 4.03 ± 0.51 out of 5, and the mean score for patient safety activities was 4.29 ± 0.49 out of 5. By occupation, the perception of PSC was 3.94 ± 0.52 among RNs and 4.15 ± 0.47 among NAs. The mean patient safety activities score was 4.46 ± 0.35 among RNs and 4.08 ± 0.56 among NAs.
3. Patient safety activities according to demographic and work-related characteristics

**Table 1:** Demographic and work related characteristics of participants (*N* = 159)

Characteristicsf	Categories	n (%)	M ± SD (range)
Occupation	Nurse aides (NAs)	71 (44.7)	
	Registered nurse (RNs)	88 (55.3)	
Sex	Men	2 ( 1.3)	
	Women	157 (98.7)	
Age (years)	< 40	18 (11.3)	50.92 ± 8.47 (25.00–69.00)
	40–49	40 (25.2)	
	50–59	79 (49.7)	
	60 ≤	22 (13.8)	
Marital status	Single	86 (54.1)	
	Married	73 (45.9)	
Education level	High school	40 (25.8)	
	Diploma/associate	50 (31.4)	
	Bachelor	52 (32.7)	
	Graduate	16 (10.1)	
Working experience at current facility (years)	< 1	51 (32.1)	51.91 ± 56.79 (0.25–25.67)
	1–4	55 (34.6)	
	5–9	32 (20.1)	
	10 ≤	21 (13.2)	
Work shift type	2 shifts	21 (13.2)	
	3 shifts	68 (42.8)	
	Fixed day shift	44 (27.7)	
	Others	26 (16.3)	
Experiences of reporting safety incident in a year	No	43 (27.0)	
	Yes	116 (73.0)	
Reported a safety incident in a year ^a^	Medication error	25 (15.7)	
	Infection	20 (12.6)	
	Fall	109 (68.6)	
	Pressure ulcer	60 (37.7)	
	Burn	13 ( 8.2)	
	Others	3 ( 1.9)	
Safety incident prevention education in a year	No	10 ( 6.3)	
	Yes	149 (93.7)	
Facility admission capacity (beds)	< 100	47 (29.5)	151.69 ± 74.23 (25.00–296.00)
	100–199	64 (40.3)	
	200 ≤	48 (30.2)	
Mean number of residents assigned per a nursing staff (person)	< 50	78 (49.1)	
	50–99	46 (28.9)	75.54 ± 63.57 (10.00–296.00)
	100 ≤	35 (22.0)	
Facility evaluation rating	A	119 (74.8)	
	B	20 (12.6)	
	C	20 (12.6)	
Ownership	Public	53 (33.3)	
	Foundation	54 (34.0)	
	Private	52 (32.7)	
Proportion of RN (%)^b^	< 25	22 (13.8)	
	25–49	33 (20.8)	
	50–74	75 (47.2)	
	75 ≤	29 (18.2)	

*From*: Predictors of Patient Safety Activities among Registered Nurses and Nurse Aides in Long-term Care Facilities

*M* Mean, *SD* Standard deviation

^a^ multiple responses. ^b^ Proportion of RNs = RNs/(RNs + NAs) × 100

**Table 2:** Level of patient safety culture and patient safety activities

Variables	Total (*N* = 159)		RN (*n* = 88)		NA (*n* = 71)	
	**M ± SD**	**Min–Max**	**M ± SD**	**Min–Max**	**M ± SD**	**Min–Max**
**Patient safety culture**	4.03 ± 0.51	2.11–5.00	3.94 ± 0.52	2.11–4.81	4.15 ± 0.47	3.19–5.00
Leadership of manager	4.02 ± 0.61	1.44–5.00	3.94 ± 0.63	1.44–5.00	4.12 ± 0.58	2.33–5.00
Work attitude	4.35 ± 0.53	2.55–5.00	4.28 ± 0.54	2.50–5.00	4.44 ± 0.50	3.17–5.00
Organizational system	4.16 ± 0.55	2.29–5.00	4.04 ± 0.55	2.29–5.00	4.30 ± 0.52	3.00–5.00
Managerial practice	3.50 ± 0.74	1.20–5.00	3.39 ± 0.74	1.20–4.80	3.64 ± 0.73	1.60–5.00
**Patient safety activities**	4.29 ± 0.49	2.80–5.00	4.46 ± 0.35	3.57–5.00	4.08 ± 0.56	2.80–5.00
Safety medication	4.05 ± 0.64	1.75–5.00	4.18 ± 0.54	2.13–5.00	3.90 ± 0.73	1.75–5.00
Infection prevention	4.30 ± 0.61	2.17–5.00	4.48 ± 0.45	3.17–5.00	4.08 ± 0.70	2.17–5.00
Fall prevention	4.38 ± 0.52	2.69–5.00	4.53 ± 0.40	3.46–5.00	4.19 ± 0.60	2.69–5.00
Pressure ulcer prevention	4.46 ± 0.71	1.00–5.00	4.75 ± 0.35	3.80–5.00	4.11 ± 0.87	1.00–5.00
Equipment/fire inspection	4.26 ± 0.73	2.33–5.00	4.44 ± 0.65	2.33–5.00	4.04 ± 0.77	2.33–5.00

*From*: Predictors of Patient Safety Activities among Registered Nurses and Nurse Aides in Long-term Care Facilities

*M* Mean, *SD* Standard deviation, *Min* Minimum value, *Max* Maximum value, *RN* Registered nurse, *NA* nurse aide

Patient safety activities were significant for occupation (t = -5.28, *p* < .001), marital status (t = 2.47, *p* = .015), education level (t = 3.43, *p* = .019), work shift type (t = 8.09, *p* < .001), experiences of reporting safety incidents (t = -2.47, *p* = .015), mean number of assigned residents (t = 4.77, *p* = .010), facility evaluation ratings (t = 5.64, *p* = .004), and proportion of RNs (t = 3.63, *p* = .014) (see Table 3). RNs performed more patient safety activities than NAs. Employees working two shifts, three shifts, and fixed day shift cycles were more active regarding patient safety than “other” shifts (i.e., fixed night shift, on-call, 24 hours). Employees who experienced adverse patient safety incidents performed more patient safety activities than those who did not. Employees at the LTC facilities with a higher percentage of RNs than NAs and good evaluation ratings performed more patient safety activities.

**Table 3:** Comparison of patient safety activities by demographic and job related characteristics of participants (*N* = 159)

Characteristics	Categories	Patient safety activities	
		**M ± SD**	**t or F (*p*)**
Occupation	NAs	4.08 ± 0.56	- 5.28 (< .001)
	RNs	4.46 ± 0.35	
Age (years.)	< 40	4.07 ± 0.62	0.46 (.713)
	40–49	4.07 ± 0.38	
	50–59	4.19 ± 0.57	
	60 ≤	3.70 ± 0.70	
Marital status	Single	4.38 ± 0.44	2.47 (.015)
	Married	4.19 ± 0.53	
Education level	High school ^a^	4.09 ± 0.63	3.43 (.019) a < b,c,d
	Diploma/associate ^b^	4.34 ± 0.39	
	Bachelor^c^	4.39 ± 0.44	
	Graduate ^d^	4.34 ± 0.46	
Working experience at current facility (years)	< 1	4.23 ± 0.49	0.81 (.489)
	1–4	4.27 ± 0.54	
	5–9	4.40 ± 0.41	
	10 ≤	4.32 ± 0.47	
Work shift type	2 shifts ^a^	4.12 ± 0.72	8.09 (< .001) a,b,c > d
	3 shifts ^b^	4.03 ± 0.58	
	Fixed day shift ^c^	4.20 ± 0.62	
	Others ^d^	3.80 ± 0.72	
Experiences of reporting safety incident in a year	No	4.13 ± 0.54	-2.47 (.015)
	Yes	4.35 ± 0.46	
Safety incident prevention education in a year	No	4.41 ± 0.55	0.71 (.494)
	Yes	4.28 ± 0.49	
Facility admission capacity (beds)	< 100	4.21 ± 0.58	1.59 (.207)
	100–199	4.28 ± 0.50	
	200 ≤	4.39 ± 0.36	
Mean number of residents assigned per a nursing staff (person)	< 50 ^a^	4.17 ± 0.51	4.77 (.010) a < c
	50–99 ^b^	4.38 ± 0.51	
	100 ≤ ^c^	4.44 ± 0.34	
Facility evaluation rating	A ^a^	4.34 ± 0.47	5.64 (.004) a,b > c
	B ^b^	4.35 ± 0.46	
	C ^c^	3.95 ± 0.53	
Ownership	Public ^a^	4.41 ± 0.35	2.39 (.095)
	Foundation ^b^	4.23 ± 0.60	
	Private ^c^	4.23 ± 0.48	
Proportion of RNs (%)^e^	< 25 ^a^	4.02 ± 0.62	3.63 (.014) a < d
	25–49 ^b^	4.32 ± 0.53	
	50–74 ^c^	4.29 ± 0.45	
	75 ≤ ^d^	4.46 ± 0.35	

*From*: Predictors of Patient Safety Activities among Registered Nurses and Nurse Aides in Long-term Care Facilities

^*^ Including fixed night shift, on-call, and 24-h shift

^†^ Proportion of RNs = RNs/(RNs + NAs) × 100

*M* Mean, *SD* Standard deviation, *yrs* Years, *NA* Nurse aide, *RN* Registered nurse

^a,b,c,d^ Scheffe’s test

### Relationship between participants’ perceptions of PSC and patient safety activities

Table 4 shows the correlations between participants’ perceptions of PSC and patient safety activities. There was a significant correlation between the perception of PSC and patient safety activities (r = 0.23, *p* = 0.004). In terms of each domain of patient safety activities, the perception of PSC was significantly positively correlated with safe medication (r = 0.24, *p* = 0.002), infection prevention (r = 0.27, *p* = 0.004), fall prevention (r = 0.18, *p* = 0.021), and equipment/fire inspection (r = 0.29, *p* < 0.001). Further, in terms of each domain of the perception of PSC, patient safety activities were significantly positively correlated with work attitude (r = 0.30, *p* < 0.001) and organizational system (r = 0.26, *p* < 0.001).

**Table 4:** Correlation coefficients of patient safety culture and patient safety activities (N = 159)

Variables	Patient safety culture r (*p*)	Subcategories			
		**Leadership of manager r (*p*)**	**Work attitude r (*p*)**	**Organizational system r (*p*)**	**Managerial practice r (*p*)**
Patient safety activities	.23 (.004)	.13 (.099)	.30 (< .001)	.26 (< .001)	.11 (.179)
Safety medication	.24 (.002)	.17 (.032)	.32 (< .001)	.26 (.001)	.11 (.181)
Infection prevention	.23 (.004)	.17 (.035)	.28 (< .001)	.24 (< .003)	.10 (.191)
Fall prevention	.18 (.021)	.08 (.313)	.26 (.001)	.23 (.004)	.09 (.262)
Pressure ulcer prevention	-.05 (.509)	-.07 (.394)	.03 (.682)	-.01 (.982)	-.12 (.134)
Equipment/fire inspection	.29 (< .001)	.16 (.049)	.27 (.001)	.28 (< .001)	.30 (< .001)

*From*: Predictors of Patient Safety Activities among Registered Nurses and Nurse Aides in Long-term Care Facilities

### Factors influencing patient safety activities

Multiple regression was performed using variables identified as predictors of patient safety activities in RNs and NAs in previous studies and variables that significantly differed in the univariate analysis in our study to identify the predictors of patient safety activities among RNs and NAs in LTC facilities. We included the domains of PSC, manager leadership, work attitude, organizational system, and managerial practice into the regression. Although education level significantly differed in the univariate analysis, it was excluded in the regression analysis. Considering that all NAs were high school graduates, there is a strong correlation between education level and occupation. Nominal variables were all dummy-coded. Multicollinearity, residuals, and outliers were assessed to test the assumption of regression for the independent variables, with all satisfying the criteria. Therefore, the regression model generated in this study was found to satisfy all assumptions of the regression equation.

Table 5 shows the results of the analysis of the predictors of patient safety activities among RNs and NAs in LTC facilities. The predictors of participants’ patient safety activities were as follows: RNs (*β* = 0.377, *p* < 0.001), the organizational safety system domain of PSC (*β* = 0.314, *p* = 0.010), and “other” work shift type (i.e., fixed night shift, on call, 24-h shift; *β* = -0.264, *p* = 0.004). These variables explained 36.0% of patient safety activities in LTC facilities (F = 5.69, *p* < 0.001).

**Table 5:** Factors influencing patient safety activities (*N* = 159)

Variables	B	SE	β	t	*p*	Multi-collinearity statistics	
						**Tolerance**	**VIF**
(Constant)	2.17	.50		4.35	< .001		
Age	.00	.00	.072	1.03	.306	.83	1.21
Marital status (ref: single)	-.12	.06	-.147	-1.92	.057	.69	1.45
Working experience at current facility	.00	.00	.106	1.38	.170	.69	1.45
Work shift type (ref: 3 shifts)							
2 shifts	-.02	.11	-.011	-0.14	.887	.72	1.39
Fixed day shift	-.00	.10	-.002	-0.02	.985	.47	2.14
**Others** ^a^	-.36	.12	-.264	-2.95	**.004**	.51	1.97
Nurses’ proportion (%)	-.00	.00	-.149	-1.40	.163	.36	2.77
Facility admission capacity	.00	.00	.057	0.48	.634	.28	3.56
Mean number of residents assigned per a nursing staff	-.00	.01	-.010	-0.11	.916	.43	2.32
Ownership (ref: public)							
Foundation	-.02	.13	-.016	-0.12	.901	.26	3.87
Private	.02	.13	.018	0.15	.884	.26	3.85
Facility evaluation rating (ref: A)							
B	.02	.12	.014	0.18	.861	.68	1.49
C	-.13	.11	-.088	-1.15	.254	.69	1.45
Experiences of reporting safety incident in a year (ref: no)	.12	.08	.109	1.49	.140	.76	1.32
**Occupation_RN** (ref: NA)	.37	.08	.377	4.64	** < .001**	.62	1.63
Subcategories of patient safety culture							
Leadership of manager	-.04	.08	-.050	-0.51	.614	.41	2.43
Work attitude	.13	.10	.137	1.23	.222	.33	3.04
**Organizational system**	.28	.11	.314	2.61	**.010**	.28	3.55
Managerial practice	-.03	.06	-.047	-0.52	.605	.50	1.99
R^2^ = .44, Adjusted R^2^ = .36, F = 5.69, *p* < .001							

*From*: Predictors of Patient Safety Activities among Registered Nurses and Nurse Aides in Long-term Care Facilities

*SE* Standard errors, *VIF* Variance inflation factors, *RN* Registered nurse, *NA* Nurse aide

^a^ Others: fixed night shift, on-call, 24-h

## Discussion

This study aimed to explore the factors that influence patient safety activities of RNs and NAs working in LTC facilities and to identify the relationship between PSC and patient safety activities. Our results found that RNs have the most important influence on patient safety activities compared to NAs and the level of patient safety activities of RNs and NAs showed significant differences. Hence, as health professionals providing bedside care for residents in LTC facilities, RNs are key personnel in charge of residents’ health and safety management [16]. A study by Shin and Hyun [19] on Korea's LTC facilities showed that increasing RN care time per resident, compared to other nursing staff, yielded better quality of care (e.g. preventing falls, decreasing tube feeding, and managing aggressive behavior). In addition, a study by Bostick et al. (2006) [17], which systematically analyzed 87 government documents published from 1975 to 2003 in the United States, found that a higher number of RNs in a LTC facility corresponded to greater improvement in resident outcomes (e.g. functional availability, pressure ulcers, weight loss). Hence, the proportion of RNs was emphasized as the most important factor in patient safety activities in LTC facilities [34].

These studies showed that replacing RNs with NAs is extremely unreasonable, as there is a substantial gap in education and qualification. Therefore, the current article of the Welfare of Senior Citizens Act stipulating that RNs and NAs are at an equivalent level, without distinguishing their qualifications, must be amended to better ensure safety and quality of care for LTC facility residents. In addition, the proportion of RNs employed at LTC facilities in Korea is 0.1%, significantly lower compared to other countries (e.g., United States = 34.3%, Netherlands = 28.2%, Germany = 50.9%, Japan = 20.7%) [35]. Due to the low standards for RNs, more than seven out of ten facilities have no RNs at all [9, 22]. Among our participants, 73.6% had reported a safety incident in the past year. These results show the seriousness of residents’ safety at LTC facilities in Korea [6]. Therefore, to reduce adverse events in residents in LTC facilities, the mandatory placement of RNs to assess and effectively manage residents at risk should be legally stipulated [16, 17, 21]. Korea has 4.2 RNs per 1,000 residents, which is lower than the OECD average of 7.9 [36]; and only 52% of RNs are active, so there is a shortage of RNs [37]. Modification of the current RNs’ staffing standard for LTC facilities in Korea is also essential for comprehensive management of safety quality for the LTC facility residents.

PSC influences employee attitudes and behaviors, regarding adherence to patient safety regulation and the practice of patient safety activities within the organization [22]. In this study, an organizational safety system of PSC was a second predictor of patient safety activities. This agrees with a previous finding that system factors, including organizational factors, are more important than individual factors in PSC. The purpose of such patient safety reporting systems is to alter the learning culture to allow staff members to learn from their failures by identifying the cause of safety incidents and apply this knowledge to practice. The most important aspect of learning from experience is to establish an organizational culture with an open reporting system, including actual adverse events and near misses [38]. An effective reporting system identifies safe behaviors that should be adapted to prevent errors, encompasses the individuals’ adherence to their safety responsibilities, promises to maintain patient safety, endeavors to acquire the latest knowledge on patient safety, and learns from errors to achieve safety goals [39]. Further, noting that administering a patient safety education program to LTC facility staff led to a reduction of potential safety incidents (e.g., falls and pressure ulcers) by increasing the staff’s awareness of PSC [40], establishing a standardized educational system for LTC facility staff is crucial. Thus, teamwork and personnel management founded on open communication, trust, and cooperation within the organization are warranted [27].

Per the recently enacted Patient Safety Act, Korea has also established an external reporting system to which relevant hospitals report patient safety incidents. The “Patient Safety Reporting Learning System” was utilized as evidence for governmental policymaking and macroscopic improvement activities to enhance patient safety and quality of care by establishing and analyzing a patient safety information database, containing data electronically submitted by hospitals [41]. The reporting system for LTC facilities should also be reinforced to examine the state of safety incidents and relevant problems, based on which appropriate safety improvement activities should be launched.

In this study, the level of patient safety activities increased with increasing perception of PSC for staff in LTC facilities. This is consistent with previous evidence of a significant correlation between the employee's perception of PSC and the outcomes of patient safety activities [5, 25, 28, 42]. As noted by other research, the measurement of PSC in LTC facilities helps improve quality of care and patient safety [28]. However, recent studies indicate a difference in the perception of PSC among staff in LTC facilities [26]. RNs also perceived lower PSC in LTC facilities, which is consistent with our findings [43] with our findings. Because participants’ perceptions of PSC reflect the current level of PSC in the LTC facility, these results suggest that RNs have a more critical view of the PSC in their facilities compared to NAs. Despite the lower perception of PSC, RNs showed significantly higher levels of patient safety activities. Amid the special situation in Korea where RNs are legally considered replaceable by NAs in LTC facilities [7], these results highlight the importance of RNs, who perceive the current PSC more critically and strive to improve it. Since perceptions of PSC vary widely among staff in LTC facilities, PSC scores should be checked according to occupation for change and evaluation of PSC in LTC facilities [26]. Therefore, managers of LTC facilities should continually measure perceived patient safety among their employees and utilize the findings as the starting point for improving PSC and increasing compliance [44].

In this study, shift types were identified as a factor affecting patient safety activities of RNs and NAs in LTC facilities. The fixed night shift, 24-h rotational shift, and on-call shift had greater negative impacts on patient safety activities compared to three-days shifts. This may be attributable to the fact that staff members who work these shift types are more likely to work excessive hours, possibly contributing to fatigue. Although no previous study examined the impact of night shift or overtime on patient safety in LTC facilities, studies of acute care hospitals have observed that fatigue caused by night shift and overtime increased the incidence of medical errors, mortality, readmission rates, and the incidence of surgical complications [45, 46]. In the United States, Germany, and Japan, LTC facility staff members generally work three shifts, and staffing standards per work hour are enforced [20, 47]. However, the regulations for nursing staff in LTC facilities in Korea set a standard of 25 residents per employed RN or NA, and the nursing workforce was caring for an average of 152 residents (range: 25–296) per duty. Hence, Korea should also develop a minimum staffing allocation standard based on three shifts. Further, 24-h patient safety activities are required, as most LTC facility residents are older adults who are frail or have dementia, requiring 24-h supervision by RNs. However, additional studies are needed to pinpoint the cause underlying the impact of shift type on patient safety activities.

Our results are significant in elucidating the need to amend regulations pertinent to RN allocation standards in LTC facilities in Korea, transitioning the current work shift to a three consecutive shifts system, and establishing a safety system at the organizational level to promote resident safety in LTC facilities.

### Limitations

This study has the following limitations: First, this study conveniently sampled RNs and NAs working in 33 (1.0%) out of 3,390 nationwide LTC facilities in Korea. Therefore, the results are limited in being representative of LTC facilities in Korea. Second, the researchers developed the patient safety activity tool based on the nursing activities performed by nursing staff in LTC facilities for older adults in Korea.

## Conclusion

Our results show the importance of enhancing the quality of safety for LTC facility residents by improving the nursing staffing standard, such as requiring the presence of RNs 24 h a day, increasing staffing, and establishing an effective shift system to strengthen patient safety activities in LTC facilities. Further, our findings suggest the importance of establishing a standardized organizational system, such as patient safety-oriented leadership, an incident reporting and communication system, a facility environment that promotes the prevention of safety incidents, and safety education programs, to foster a safety culture in LTC facilities and to ensure the safety of residents.

## Supplementary Information

**Additional file 1:** **Appendix A. **Patientsafety sctivities.

**Additional file 2:** 

## Data Availability

The datasets used and/or analyzed during the current study are available from the corresponding author on reasonable request**.**

## Abbreviations

AHRQ: Agency for Healthcare Research and Quality
CVI: Content validity index
KABONE: Korea Accreditation Board of Nursing Education
LTC: Long-term care
NHIS: National Health Insurance Service
NA: Nurse aide
RN: Registered nurse
PSC: Patient safety culture
