# Correction: Predictors of patient safety activities among registered nurses and nurse aides in long-term care facilities: cross-sectional study

**DOI:** 10.1186/s12877-022-03264-4

**Published:** 2022-07-19

**Authors:** Youran Lee, Eunhee Cho

**Affiliations:** 1 Duke University School of Nursing, 307 Trent Drive, Durham, NC 27710 USA; 2 Mo-Im Kim Nursing Research Institute, Yonsei University College of Nursing, 50-1 Yonsei-ro, Seodaemoon-Gu, Seoul, 03722 South Korea

**Correction; BMC Geriatrics (2022) 22:541**

**https://doi.org/10.1186/s12877-022-03234-w**

After publication of this article [1], it was noted that erroneously 2 additional files were published. These have been removed and the original article [1] has been corrected.

## References

[1] Lee Y, Cho E (2022). Predictors of patient safety activities among registered nurses and nurse aides in long-term care facilities: cross-sectional study. BMC Geriatrics.

