# Supplementary material for: Predictors of patient safety activities among registered nurses and nurse aides in long-term care facilities: cross-sectional study
Source: BMC Geriatr. 2022 Jun 29;22:541. doi: 10.1186/s12877-022-03234-w (PMC9245202; doi:10.1186/s12877-022-03234-w)
Supplement: Supplementary file 1 — Additional file 1: Appendix A. Patientsafety sctivities. [file 12877_2022_3234_MOESM1_ESM.docx]

**Appendix A: Patient Safety Activities**

The following statements describe how you feel about your patient safety activities. Please, indicate how strongly you agree on each statement. (1=strongly disagree, 2=disagree, 3=not sure, 4=agree, 5=strongly agree)

| **Safe medication** | | | | | | |
| --- | --- | --- | --- | --- | --- | --- |
| Q1. | I use at least two resident identifiers when administering medication (e.g. name and date of birth). | 1 | 2 | 3 | 4 | 5 |
| Q2. | I check ‘five rights’ every time when administering medications to the resident. | 1 | 2 | 3 | 4 | 5 |
| Q3. | I know precautions for high-risk medications (e.g. heparin, warfarin, insulin, etc.) and how to respond to side effects. | 1 | 2 | 3 | 4 | 5 |
| Q4. | I check the blood test results of the resident before administer the anticoagulant (e.g. INR, aPTT). | 1 | 2 | 3 | 4 | 5 |
| Q5. | I provide education on anticoagulant therapy to residents and their families who are taking anticoagulants (e.g. side effect, possible drug interaction, etc.). | 1 | 2 | 3 | 4 | 5 |
| Q6. | I record the correct information about the medications being administered to the resident (e.g., drug name, dosage, path, frequency, duration, purpose, etc.). | 1 | 2 | 3 | 4 | 5 |
| Q7. | I collect information about the medications that the resident is taking at the time of admission and compare them with the drugs that are newly administered after admission. | 1 | 2 | 3 | 4 | 5 |
| Q8. | I explain to residents and caregivers the effects, dosage, cautions and so on of the medication being taken. | 1 | 2 | 3 | 4 | 5 |
| **Fall prevention** | | | | | | |
| Q1. | I assess the fall risk using the fall risk assessment tool on resident admission. | 1 | 2 | 3 | 4 | 5 |
| Q2. | I educate caregiver on the fall reduction program of fall risk resident. | 1 | 2 | 3 | 4 | 5 |
| Q3. | I always raise bedside rails when moving bed. | 1 | 2 | 3 | 4 | 5 |
| Q4. | I completely tight wheelchair brake when moving a resident into or out of wheelchair.. | 1 | 2 | 3 | 4 | 5 |
| Q5. | I respond immediately when the bedside bell rings. | 1 | 2 | 3 | 4 | 5 |
| Q6. | I take action to my residents to go to the bathroom before going to bed. | 1 | 2 | 3 | 4 | 5 |
| Q7. | I leave the bed light on in the resident's rooms at night. | 1 | 2 | 3 | 4 | 5 |
| Q8. | I leave the hallway light on during night shift. | 1 | 2 | 3 | 4 | 5 |
| Q9. | I use signage to identify those residents who are 'known fallers or risk fallers'. | 1 | 2 | 3 | 4 | 5 |
| Q10. | I regularly check the wheels of beds and wheelchairs. | 1 | 2 | 3 | 4 | 5 |
| Q11. | I educate transport personal on any individualized fall reduction strategies. | 1 | 2 | 3 | 4 | 5 |
| Q12. | I reevaluate the fall risk when resident have changing status and risk drug taken. | 1 | 2 | 3 | 4 | 5 |
| Q13. | I evaluate the effectiveness of all fall reduction activities, including assessment, interventions and education. | 1 | 2 | 3 | 4 | 5 |

| **Infection prevention** | | | | | | |
| --- | --- | --- | --- | --- | --- | --- |
| Q1. | I conduct hand hygiene before contact or treatment with a resident. | 1 | 2 | 3 | 4 | 5 |
| Q2. | I conduct hand hygiene after contacting the residents and their surroundings. | 1 | 2 | 3 | 4 | 5 |
| Q3. | I conduct hand hygiene after contact with the resident's blood or body fluids. | 1 | 2 | 3 | 4 | 5 |
| Q4. | I wear personal protective equipment when nursing residents with infectious diseases. | 1 | 2 | 3 | 4 | 5 |
| Q5. | I isolate and care a resident with infectious diseases (e.g. multidrug-resistant organisms, tuberculosis, etc.). | 1 | 2 | 3 | 4 | 5 |
| Q6. | I periodically measure and monitor the infectious diseases prevention process and outcomes in the residents. | 1 | 2 | 3 | 4 | 5 |
| Q7. | I educate caregivers about prevention and management strategies of infectious disease in the residents. | 1 | 2 | 3 | 4 | 5 |
| Q8. | I follow written procedures based on established evidence-based practice guidelines for inserting and maintaining an indwelling urinary catheter. | 1 | 2 | 3 | 4 | 5 |
| Q9. | I educate caregivers about infection prevention and management strategies of residents with indwelling urinary catheters. | 1 | 2 | 3 | 4 | 5 |
| Q10. | I measure and monitor catheter-associated urinary tract infection prevention processes and outcomes. | 1 | 2 | 3 | 4 | 5 |
| Q11. | I separate and collect medical wastes according to the facility regulations. | 1 | 2 | 3 | 4 | 5 |
| Q12. | I separate and store contaminated laundry and other laundry according to the facility regulations. | 1 | 2 | 3 | 4 | 5 |
| **Pressure ulcer prevention** | | | | | | |
| Q1. | I perform an initial assessment at admission to identify residents at risk for pressure ulcers. | 1 | 2 | 3 | 4 | 5 |
| Q2. | I conduct a systematic risk assessment for pressure ulcers using a validated risk assessment tool such as the Braden or Norton Scales. | 1 | 2 | 3 | 4 | 5 |
| Q3. | I educate caregivers on how to identify risk for and prevent pressure ulcers. | 1 | 2 | 3 | 4 | 5 |
| Q4. | I take action to address any identified risks to the resident for pressure ulcers, | 1 | 2 | 3 | 4 | 5 |
| Q5. | I reassess and reevaluate pressure ulcer risk at intervals defined by the organization. | 1 | 2 | 3 | 4 | 5 |
| **Equipment/fire inspection** | | | | | | |
| Q1. | I regularly check the call bell and various alarm systems, and request immediate repair on failure. | 1 | 2 | 3 | 4 | 5 |
| Q2. | I regularly check medical devices and equipment used in the facility. | 1 | 2 | 3 | 4 | 5 |
| Q3. | I am familiar with the fire prevention plan and the fire protection plan defined by the organization. | 1 | 2 | 3 | 4 | 5 |
